# Diosmin Attenuates PTSD-like Behaviors and Hippocampal Astrocyte Alterations with Reduced STAT1 and CXCL9 Expression in an SPS&S Mouse Model

**DOI:** 10.3390/ijms27209342

**Published:** 2026-10-21

**Authors:** Xinchao Zhang, Mengjie Ren, Anke Xu, Jintian Liu, Dongran Liang, Fang Kuang, Mingmei Wu

**Affiliations:** 1 Department of Neurobiology, School of Basic Medicine, Fourth Military Medical University, Xi’an 710032, China; 17355902055@163.com (X.Z.);; 2 The Shaanxi Province Key Laboratory of Brain Function Analysis and Modulation, Xi’an 710032, China

**Keywords:** post-traumatic stress disorder, single prolonged stress and shock, diosmin, astrocytes, neuroinflammation, STAT1, CXCL9, conditioned fear

## Abstract

Diosmin is a citrus-derived flavone glycoside with anti-inflammatory activity in preclinical models. This study aimed to investigate the effects of diosmin on PTSD-like behaviors, astrocyte morphology, and the expression of STAT1 and CXCL9 in a single prolonged stress and shock (SPS&S) mouse model, and to assess whether *Stat1* knockdown could mimic diosmin’s effects in primary astrocytes. Male C57BL/6J mice were assigned to the control, SPS&S, and SPS&S + diosmin groups and underwent 14 days of treatment. Behavioral assessments included open-field and elevated plus-maze tests, alongside cue- and context-associated fear tests, complemented by GFAP-based morphometry, immunofluorescence, Western blotting, serum cytokine assays, hippocampal RNA sequencing, and quantitative reverse-transcription PCR (qRT-PCR). The results indicated that diosmin significantly enhanced exploratory behavior and reduced fear responses compared to SPS&S alone, alongside notable alterations in hippocampal astrocyte morphology and a shift in cytokine profiles, with decreased pro-inflammatory cytokines and increased anti-inflammatory markers. RNA sequencing revealed 297 differentially expressed genes, particularly highlighting the roles of *Stat1* and *Cxcl9,* both of which were significantly downregulated upon diosmin treatment. Furthermore, diosmin modulated astrocyte responses to inflammatory stimuli in primary cultures. These findings suggest that diosmin exerts protective effects against PTSD-like symptoms by regulating astrocytic function and immune-related pathways, with STAT1 signaling serving as a key but non-exclusive downstream mechanism underlying its therapeutic actions. Future studies should further elucidate the full spectrum of associated pathways to advance the development of targeted interventions for PTSD.

## 1. Introduction

Post-traumatic stress disorder (PTSD) is a trauma- and stressor-related disorder characterized by intrusive recollections, avoidance, negative alterations in cognition and mood, and hyperarousal. Its clinical heterogeneity and incomplete response to current therapies motivate investigations of biological processes that may sustain persistent fear- and stress-related behavior [1,2,3].

The pathophysiology of PTSD is complex and remains incompletely understood, yet emerging evidence underscores the critical roles of neuroinflammation and glial dysfunction involving astrocytes and microglia [4,5,6,7]. Specifically, astrocytes, the most abundant glial cells in the central nervous system, are increasingly recognized as key contributors to the pathophysiology of stress-related disorders [8,9,10]. Under chronic stress, astrocyte morphology, molecular programs, and imaging markers can be altered. Studies have demonstrated that chronic stress induces astrocytic reactivity and a reduction in the number and complexity of hippocampal astrocytes, which is associated with impaired synaptic plasticity and cognitive deficits [10,11]. Furthermore, the neurotoxic astrocyte has been shown to contribute to the loss of synaptic function and neuronal damage [12,13]. Accumulating studies indicate that modulating the toxic astrocytes can exert neuroprotective effects [14,15,16,17]. The convergence of astrocytic dysfunction, phenotypic changes, and neuroinflammation in the hippocampus thus represents a critical mechanism in the pathogenesis of PTSD, underscoring the need for therapeutic strategies that target these interconnected pathways. However, effective pharmacological interventions capable of modulating this astrocytic phenotypic switch to achieve both anti-inflammatory and neuroprotective effects concurrently remain largely unexplored.

Diosmin is a citrus-derived flavonoid that exhibits anti-inflammatory and neuroprotective effects in multiple preclinical models. Previous studies have documented its behavioral and molecular activities in models of lipopolysaccharide (LPS)-induced depressive-like behavior, traumatic brain injury, and Alzheimer’s disease [18,19,20,21,22]. Although diosmin ameliorates LPS-induced depression-like behaviors in mice by inhibiting inflammation and oxidative stress in the prefrontal cortex [23], it remains unclear whether diosmin modulates PTSD-like behaviors concomitant with hippocampal astrocyte-associated alterations.

In this study, we evaluated the effects of diosmin in male mice subjected to single prolonged stress and shock (SPS&S). We assessed anxiety-associated exploratory behaviors and conditioned freezing responses, quantified GFAP-labeled morphological features together with C3 and S100A10 expression in the hippocampus, and profiled hippocampal gene expression as well as circulating inflammatory factors. Transcriptomic screening prompted a focused assessment of STAT1 and CXCL9, followed by primary astrocyte experiments using *Stat1* siRNA to test whether *Stat1* knockdown reproduced or modified selected diosmin-associated molecular effects.

## 2. Results and Discussion

### 2.1. Diosmin Attenuates Anxiety-Related Behavioral Measures and Excessive Conditioned Freezing in SPS&S-Exposed Mice

**Figure 1:**
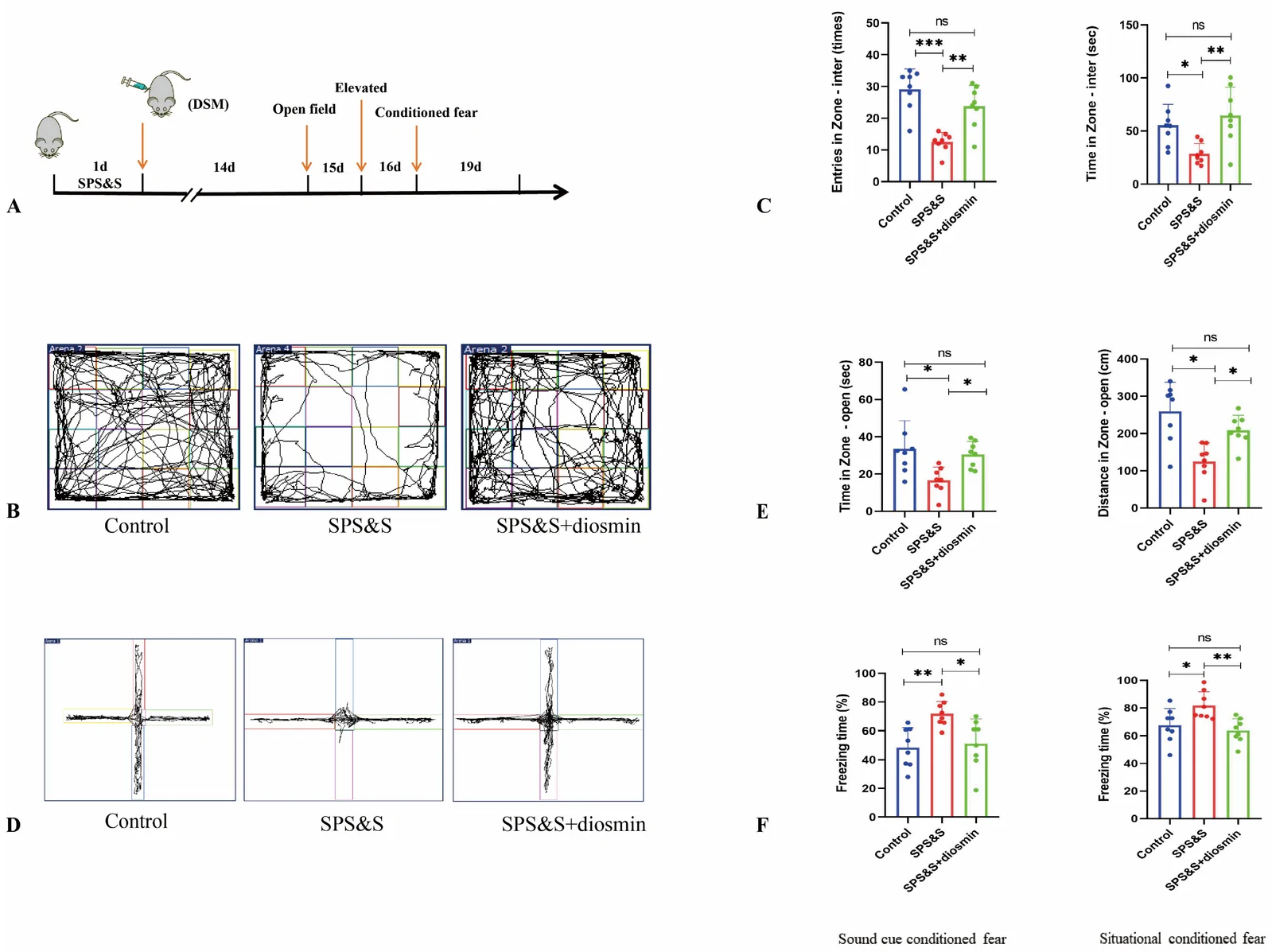
Diosmin attenuates anxiety-related exploration deficits and excessive conditioned freezing in SPS&S-exposed mice. (**A**) Experimental timeline: SPS&S on day 1, 14 consecutive days of diosmin (DSM) treatment, and behavioral testing on days 15–19. (**B**) Representative open-field trajectories. (**C**) Numbers of central-zone entries and time spent in the central zone in the OFT. (**D**) Representative EPM trajectories. (**E**) Time and distance traveled in the open arms of the EPM. (**F**) Freezing percentages during cue-associated and context-associated tests. Data are presented as mean ± SD, *n* = 8/group. * *p* < 0.05, ** *p* < 0.01, *** *p* < 0.001; ns, not significant.

In the open-field test (OFT) on day 15, SPS&S-exposed mice made fewer entries into the central zone (*p* < 0.001) and spent less time (*p* < 0.05) there than control mice. Diosmin increased both central-zone entries and central-zone time relative to the untreated SPS&S group (both *p* < 0.01); neither measure differed significantly between the SPS&S + diosmin and control groups (Figure 1B,C). In the Elevated Plus Maze (EPM) on day 16, SPS&S reduced time and distance traveled in the open arms relative to controls (both *p* < 0.05). Diosmin increased both measures relative to the untreated SPS&S group (both *p* < 0.05), and the SPS&S + diosmin group did not differ significantly from controls (Figure 1D,E).

During cue-associated testing on day 18 and context-associated no-shock exposure on day 19, SPS&S-exposed mice showed higher freezing percentages than controls (*p* < 0.01 and *p* < 0.05). Diosmin reduced freezing in both paradigms relative to the untreated SPS&S group (*p* < 0.05 and *p* < 0.01, respectively) with no significant difference between the SPS&S + diosmin and control groups (Figure 1F). Thus, diosmin attenuated excessive conditioned freezing under the reported test conditions.

### 2.2. Diosmin Partly Reverses Reductions in Hippocampal GFAP-Positive Profiles and Process-Complexity Measures

**Figure 2:**
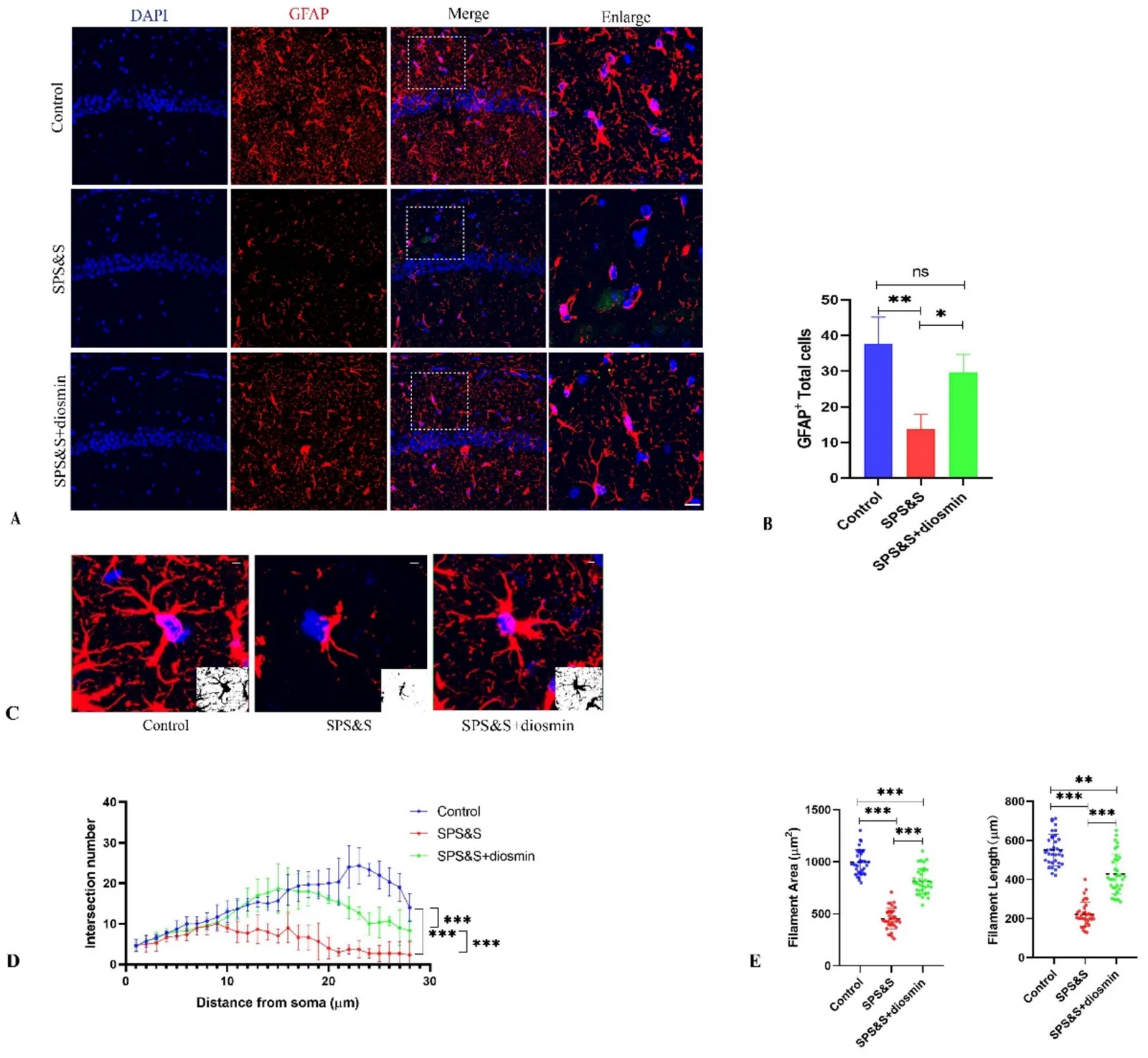
Diosmin partly reverses SPS&S-associated reductions in hippocampal GFAP immunoreactivity and GFAP-labeled process complexity. (**A**) Representative GFAP and DAPI images from the hippocampal CA1 region. Scale bar, 20 µm. (**B**) Proportion of GFAP-positive profiles among DAPI-stained cells. (**C**) Representative high-magnification GFAP-positive cells used for morphometric analysis. Scale bar, 5 µm. (**D**) Sholl analysis of process intersections as a function of radial distance from the soma. (**E**) Total filament area and total filament length of individual GFAP-positive cells. Data are presented as mean ± SD. * *p* < 0.05, ** *p* < 0.01, *** *p* < 0.001; ns, not significant.

GFAP immunofluorescence in the hippocampal CA1 region showed a lower proportion of GFAP-positive profiles among DAPI-stained cells in SPS&S-exposed mice than in controls (*p* < 0.01). This proportion was higher after diosmin treatment (*p* < 0.05) and did not differ significantly from the control group (Figure 2A,B). Single-cell GFAP-based morphometry further revealed reduced Sholl intersections, along with decreased total filament area and length, following SPS&S exposure (*p* < 0.001). Diosmin elevated these morphometric indices in SPS&S-exposed mice compared with untreated counterparts (*p* < 0.001). Several parameters nevertheless remained significantly different from control values. These observations indicate partial rather than full restoration of GFAP-labeled process complexity (Figure 2C–E).

### 2.3. Diosmin Alters Hippocampal Astrocyte-Associated Markers, Inflammatory Proteins, and Serum Cytokines

**Figure 3:**
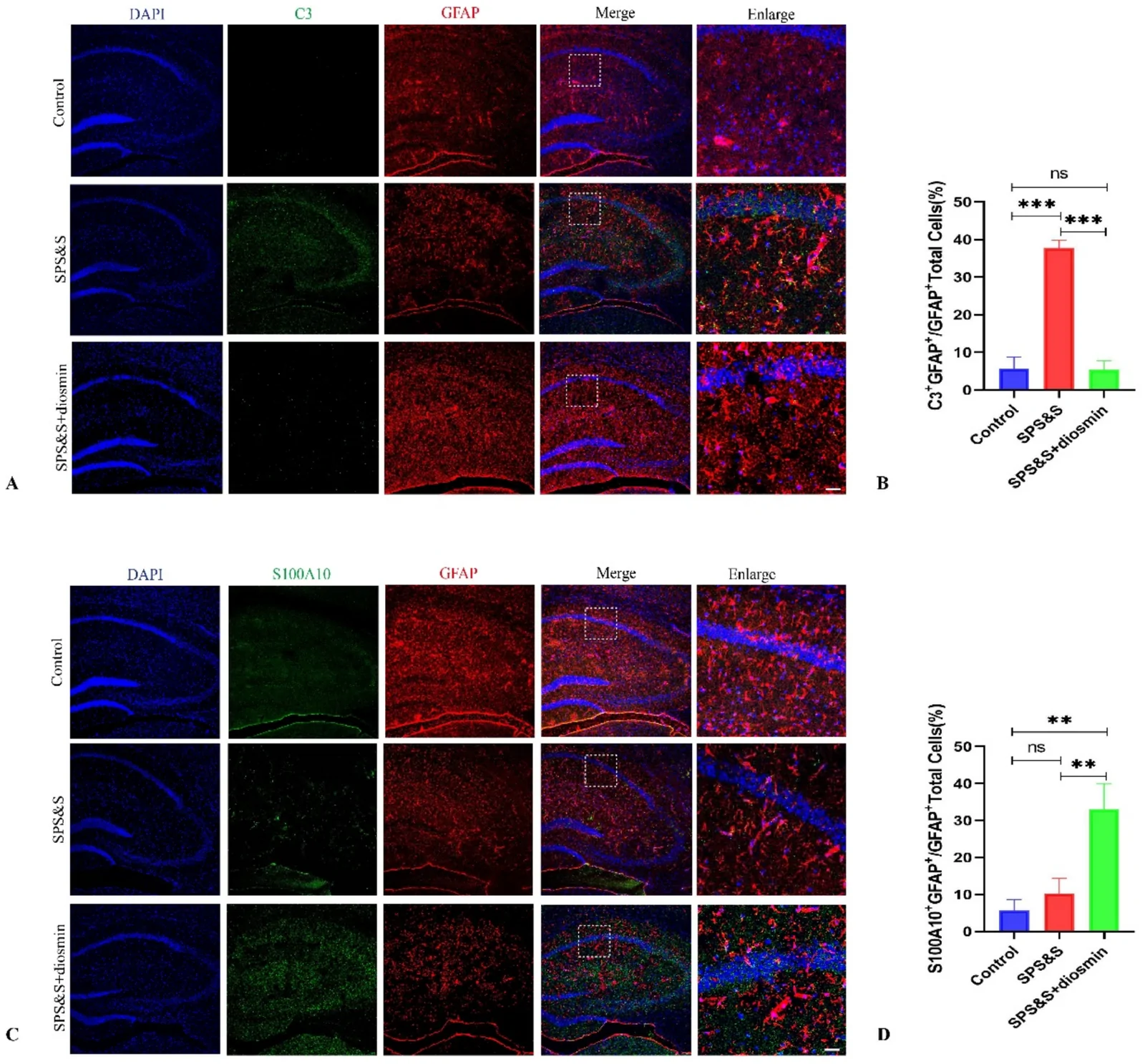
Diosmin decreases C3 co-expression and increases S100A10 co-expression in hippocampal GFAP-positive cells. (**A**) Representative DAPI, C3, and GFAP immunofluorescence in control, SPS&S, and SPS&S + diosmin groups. (**B**) Percentage of C3-positive/GFAP-positive profiles among GFAP-positive cells. (**C**) Representative DAPI, S100A10, and GFAP immunofluorescence. (**D**) Percentage of S100A10-positive/GFAP-positive profiles among GFAP-positive cells. Scale bar, 20 µm. Data are presented as mean ± SD. ** *p* < 0.01, *** *p* < 0.001; ns, not significant.

Compared with controls, SPS&S exposure increased the proportion of C3-positive/GFAP-positive profiles among GFAP-positive cells (*p* < 0.001). Diosmin reduced this proportion relative to untreated SPS&S mice (*p* < 0.001) to a value that did not differ significantly from that of the controls (Figure 3A,B). The proportion of S100A10-positive/GFAP-positive profiles did not differ significantly between control and SPS&S groups, whereas diosmin increased it relative to both groups (*p* < 0.01; Figure 3C,D).

**Figure 4:**
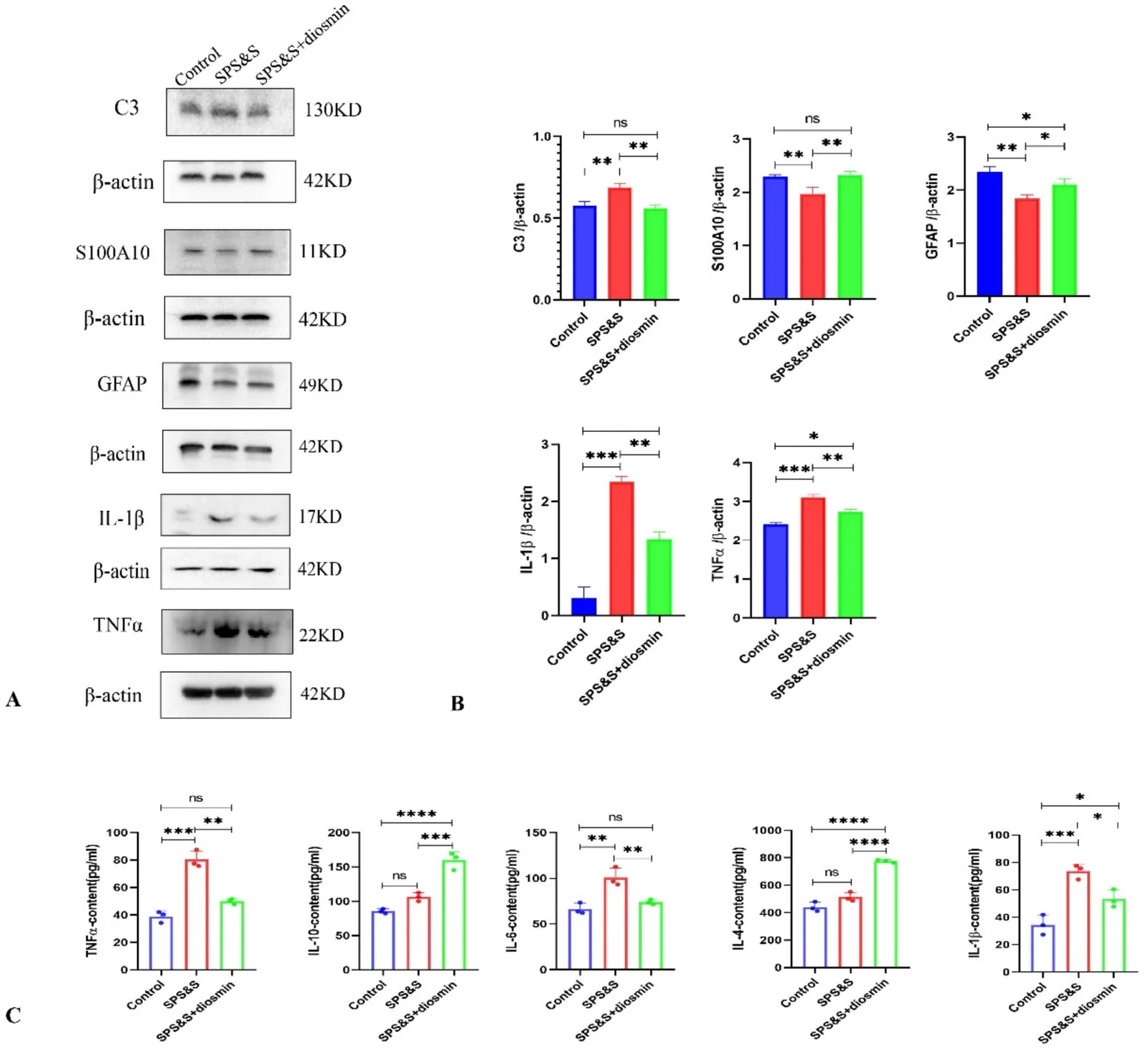
Diosmin partly reverses hippocampal protein changes and modifies serum cytokines in SPS&S-exposed mice. (**A**) Representative Western blots for C3, S100A10, GFAP, IL-1β, and TNF-α in hippocampal homogenates. (**B**) Densitometric values normalized to β-actin. (**C**) Serum TNF-α, IL-1β, IL-6, IL-4, and IL-10 measured by ELISA. Data are presented as mean ± SD. * *p* < 0.05, ** *p* < 0.01, *** *p* < 0.001, **** *p* < 0.0001; ns, not significant.

Western blotting analysis of hippocampal homogenates demonstrated elevated expression levels of C3 (*p* < 0.01), IL-1β (*p* < 0.001), and TNF-α (*p* < 0.001), along with decreased S100A10 (*p* < 0.01) and GFAP (*p* < 0.01) in SPS&S-exposed mice relative to control mice. Diosmin treatment significantly reversed these aberrant expression patterns (GFAP, *p* < 0.05, other four indicators, *p* < 0.01) and restored the levels of all five indicators close to the control levels. Nevertheless, the expression of GFAP, IL-1β, and TNF-α still maintained significant differences between the diosmin-treated group and the control group according to quantitative analysis. These results suggested that diosmin only partially normalized the abnormal molecular alterations induced by SPS&S, rather than achieving complete recovery (Figure 4A,B). In serum samples, SPS&S exposure markedly upregulated the levels of pro-inflammatory TNF-α (*p* < 0.001), IL-1β (*p* < 0.001) and IL-6 (*p* < 0.01). No significant differences were observed in the anti-inflammatory cytokines IL-4 and IL-10 between the SPS&S group and the control group. Diosmin effectively reduced the elevated serum levels of the three pro-inflammatory cytokines (*p* < 0.01, *p* < 0.05, and *p* < 0.01) in SPS&S mice. It also further upregulated IL-4 (*p* < 0.001) and IL-10 (*p* < 0.01) expression beyond the levels detected in both untreated SPS&S mice and normal controls (Figure 4C).

### 2.4. Diosmin Is Associated with Immune-Related Transcriptomic Changes and Reduced Hippocampal STAT1 and CXCL9 Expression

**Figure 5:**
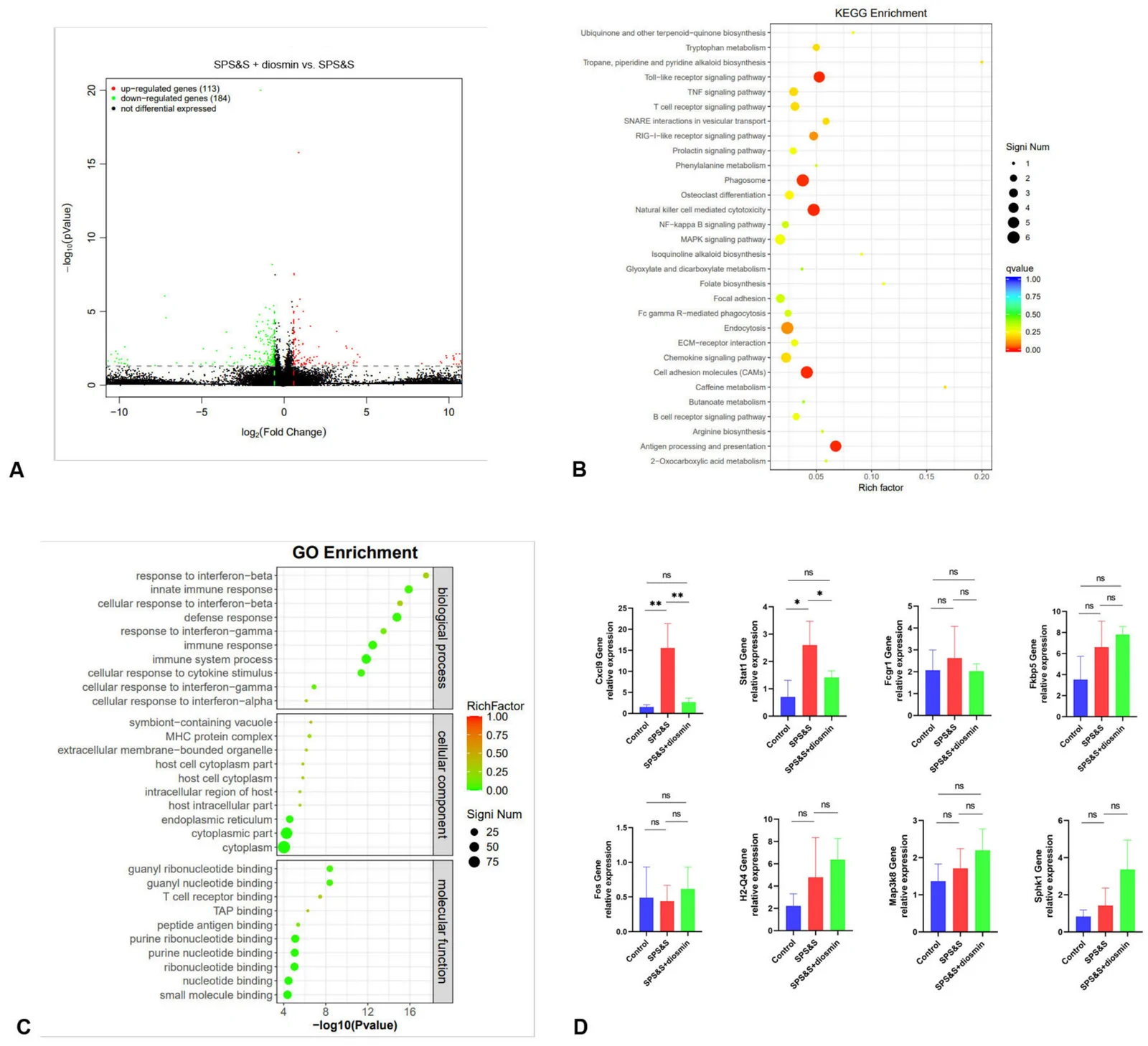
Hippocampal transcriptomic profiling and targeted qRT−PCR assessment. (**A**) Volcano plot for SPS&S + diosmin versus SPS&S, showing 113 upregulated and 184 downregulated genes. (**B**) KEGG and (**C**) GO enrichment plots. (**D**) qRT−PCR assessment of several selected genes across control, SPS&S, and SPS&S + diosmin groups; only *Cxcl9* and *Stat1* show significant group differences in the displayed analyses. Data are presented as mean ± SD. * *p* < 0.05, ** *p* < 0.01; ns, not significant.

RNA sequencing analysis was performed on hippocampal tissues from the SPS&S + diosmin and untreated SPS&S groups. A total of 297 differentially expressed genes were identified in the SPS&S + diosmin group relative to the SPS&S group, consisting of 113 upregulated genes and 184 downregulated genes (Figure 5A). Subsequent KEGG pathway and GO functional enrichment analyses revealed prominent enrichment of immune-associated biological terms. These terms mainly encompassed antigen processing and presentation, Toll-like receptor signaling, TNF signaling, chemokine signaling, and interferon-mediated responses (Figure 5B,C). Candidate gene expression was further verified via qRT-PCR. Among the detected genes, only *Cxcl9* (*p* < 0.01) and *Stat1* (*p* < 0.05) exhibited significantly elevated expression following SPS&S stimulation. Their upregulated expression was markedly reversed by diosmin treatment. The remaining six candidate genes showed no statistically significant differences across experimental groups in the validated results (Figure 5D).

**Figure 6:**
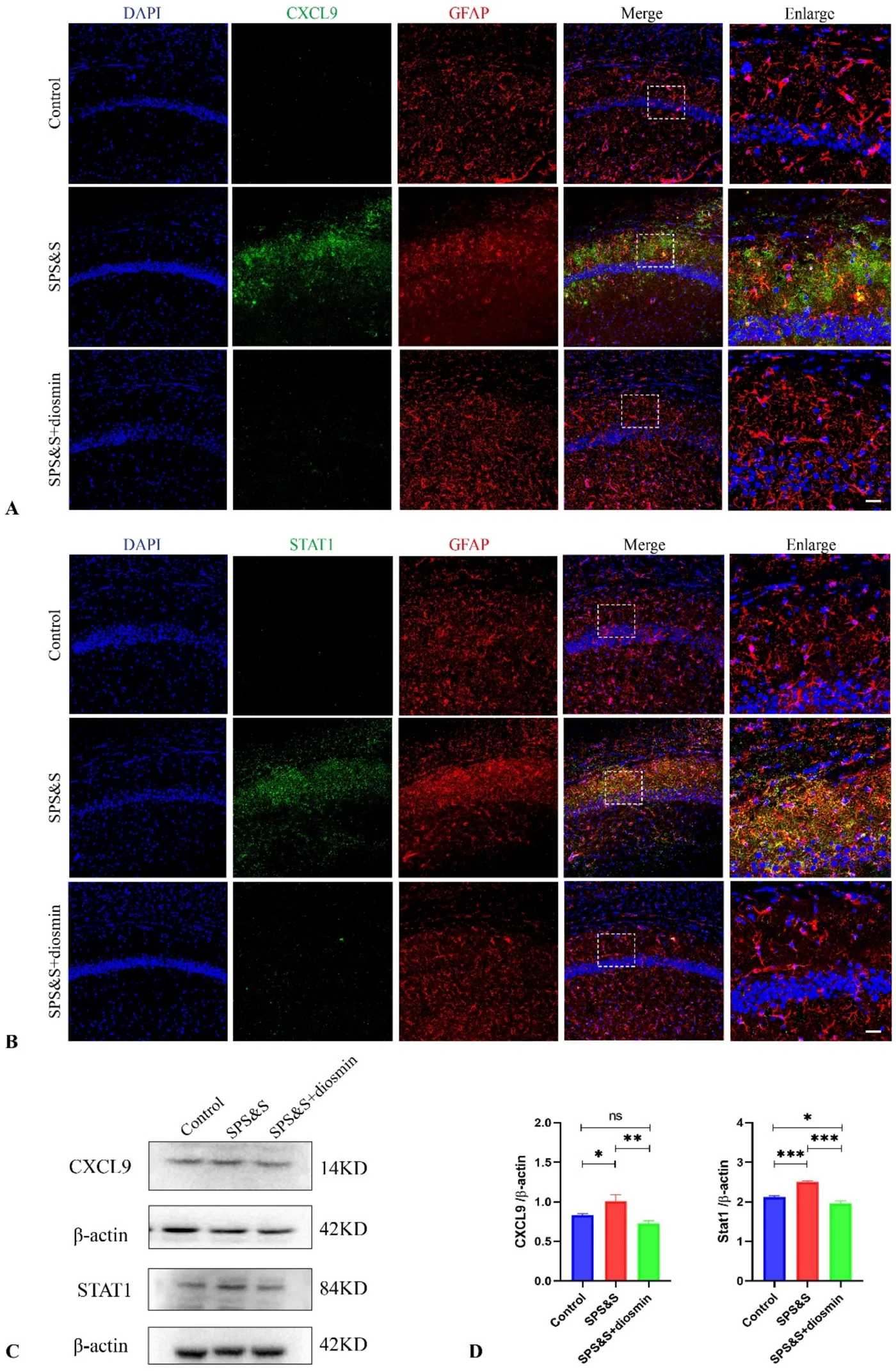
Diosmin reduces hippocampal STAT1 and CXCL9 expression in SPS&S-exposed mice. (**A**,**B**) Representative DAPI/CXCL9/GFAP and DAPI/STAT1/GFAP immunofluorescence. Scale bar, 20 µm. (**C**) Representative Western blots of total CXCL9 and STAT1 in hippocampal homogenates. (**D**) Densitometric values normalized to β-actin. Data are presented as mean ± SD. * *p* < 0.05, ** *p* < 0.01, *** *p* < 0.001; ns, not significant.

Representative immunofluorescence images exhibited intensified CXCL9 and STAT1 signals within GFAP-labeled regions in the SPS&S group. Diosmin treatment effectively attenuated such signal alterations (Figure 6A,B). Western blotting of whole hippocampal lysates demonstrated elevated total CXCL9 (*p* < 0.05) and STAT1 (*p* < 0.001) protein levels in SPS&S mice compared with control mice. Diosmin intervention significantly decreased the abundance of these two proteins (*p* < 0.01 and *p* < 0.001, respectively; Figure 6C,D).

### 2.5. Diosmin Modifies C3 or S100A10 Under Distinct Astrocyte Induction Conditions

**Figure 7:**
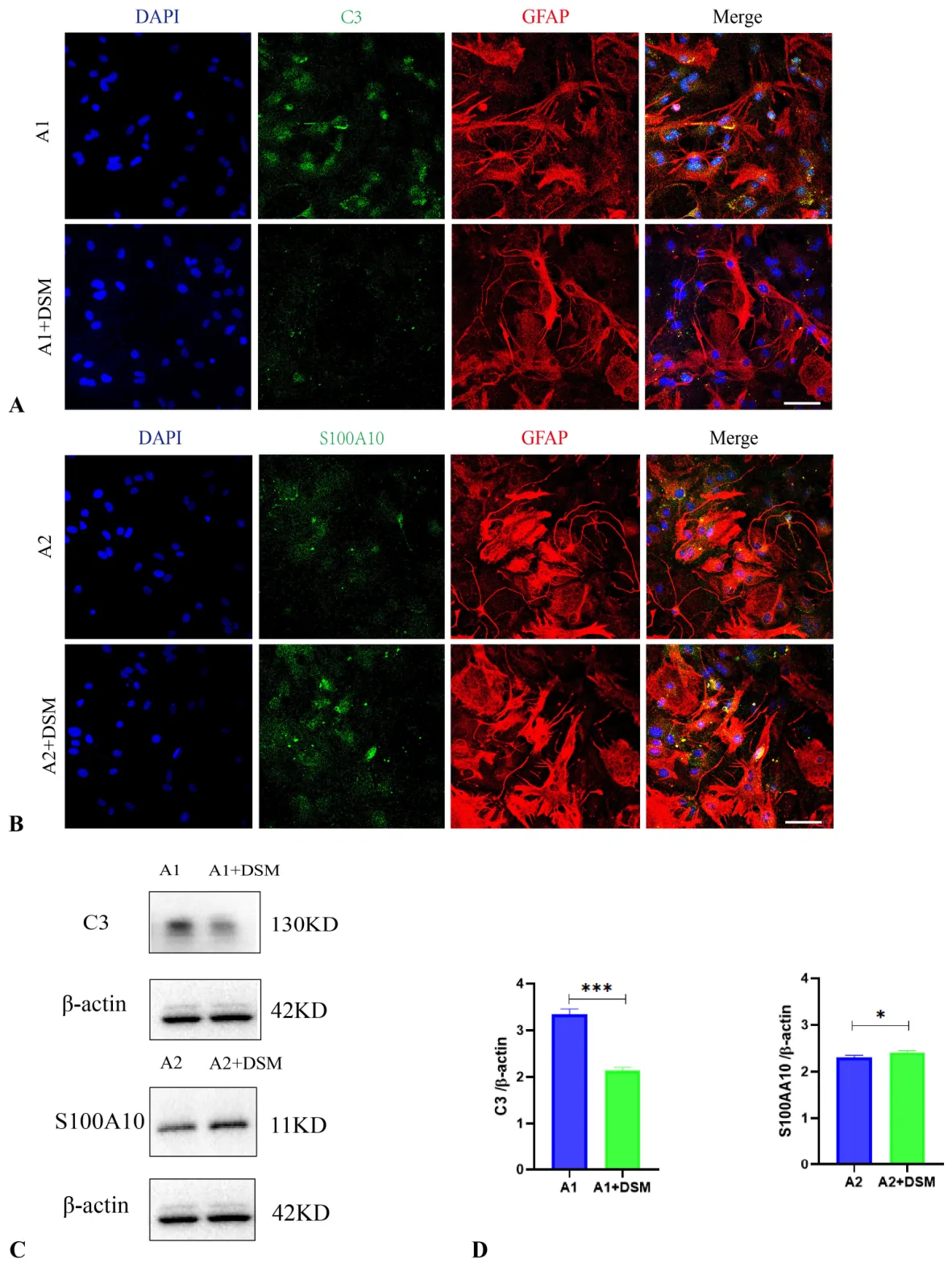
Diosmin modifies C3 and S100A10 expression under distinct primary astrocyte induction conditions. (**A**) Representative DAPI/C3/GFAP images under IL-1α/TNF-α/C1q induction with or without diosmin. (**B**) Representative DAPI/S100A10/GFAP images under IL-4/IL-10 induction with or without diosmin. Scale bar, 50 µm. (**C**,**D**) Representative Western blots and densitometric analysis of C3 or S100A10 normalized to β-actin. Data are presented as mean ± SD. * *p* < 0.05, *** *p* < 0.001.

**Figure 8:**
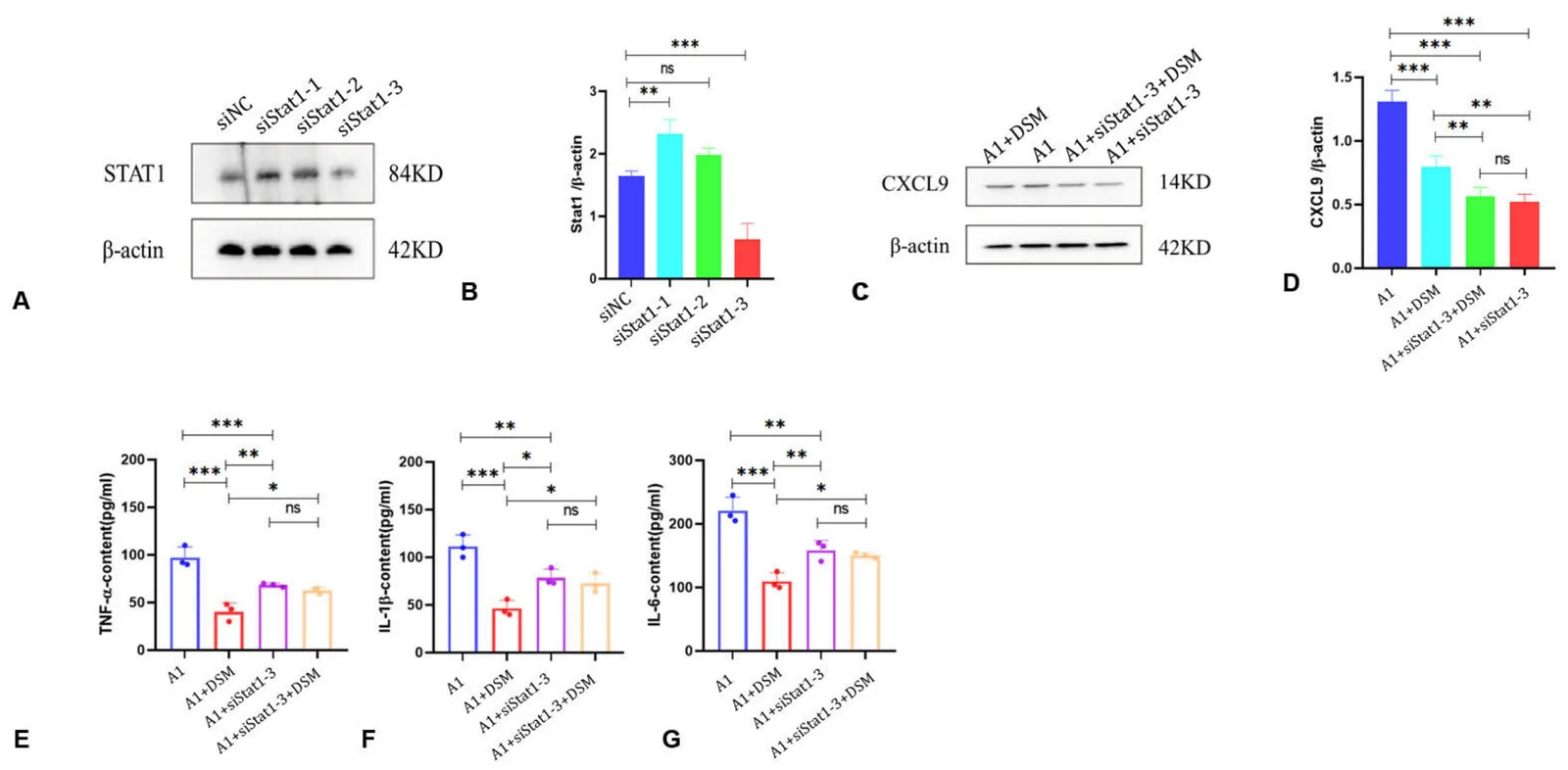
*Stat1* knockdown and diosmin reduce CXCL9 and pro-inflammatory cytokine release in induced primary astrocyte cultures. (**A**,**B**) Screening of three *siStat1* constructs; *siStat1-3* was used subsequently. (**C**,**D**) CXCL9 protein under the indicated treatments. (**E**–**G**) TNF-α, IL-1β, and IL-6 in culture supernatants. Data are presented as mean ± SD. * *p* < 0.05, ** *p* < 0.01, *** *p* < 0.001; ns, not significant.

In primary astrocyte-enriched cultures stimulated with IL-1α/TNF-α/C1q, diosmin diminished both C3 immunoreactivity and C3 protein abundance (*p* < 0.001). In parallel cultures treated with IL-4/IL-10, diosmin moderately upregulated S100A10 expression (*p* < 0.05, Figure 7A–D). Three independent *Stat1* siRNAs were screened in preliminary experiments. The *siStat1-3* construct yielded the most robust suppression of STAT1 expression and was therefore selected for all subsequent functional assays (*p* < 0.001; Figure 8A,B). In IL-1α/TNF-α/C1q-activated astrocyte cultures, either diosmin treatment or *Stat1* silencing alone significantly decreased CXCL9 protein levels relative to the stimulated control group (*p* < 0.001). Combined administration of diosmin in *Stat1*-knockdown cells failed to further reduce CXCL9 expression beyond the effect of *Stat1* silencing alone (Figure 8C,D). Both diosmin intervention and *Stat1* knockdown reduced the secretion of TNF-α, IL-1β, and IL-6 in culture supernatants (*p* < 0.01). The combination of diosmin and *Stat1* knockdown did not produce additional inhibitory effects compared with *Stat1* silencing alone (Figure 8E–G). The absence of additive effects indicates overlapping or convergent regulatory mechanisms centered on Stat1. Nevertheless, these results do not definitively verify that *Stat1* is an essential mediator for diosmin function or that CXCL9 governs the downstream cytokine responses.

### 2.6. Discussion

This study evaluated the therapeutic potential of diosmin, a flavonoid with well-documented clinical safety, in a mouse model of PTSD. Diosmin treatment yielded ameliorated behavioral outcomes corresponding to core PTSD-related symptomatology. Enhanced exploration of the EPM open arms and the OFT central region indicated reduced generalized anxiety and avoidance responses, whereas decreased freezing during contextual and cued fear conditioning pointed to mitigated pathological fear-memory retrieval and hyperarousal.

Our data demonstrate that diosmin confers robust neuroprotection to hippocampal astrocytes and reverses stress-triggered atrophy and functional impairment. Diosmin restored GFAP expression and rescued complex arborization, as quantified by Sholl analysis. These observations reflect recovered astrocytic structural integrity. Such structural deficits have been reported in preclinical PTSD models and are consistent with reduced astrocytic markers observed in human PET imaging [10,24]. Our findings support the established role of astrocytes in neuroinflammation and neurodegenerative cascades under stress conditions. Astrocyte responses vary across different pathological settings, and reactivity is not defined by hypertrophy alone [25]. Here, decreased GFAP-positive profiles and process complexity describe the labeled cytoskeleton, while C3 and S100A10 provide only limited molecular information. Changes in GFAP expression can alter process visibility and detection thresholds. These findings therefore cannot independently establish cell loss or whole-cell atrophy. As critical stress response mediators, astrocytes rapidly respond to traumatic stimuli and maintain cerebral homeostasis. Reactive astrocytes can transform into neurotoxic A1-like phenotypes with upregulated C3, TNF-α and IL-1β, which trigger synaptic dysfunction and neuronal injury [26]. Single-cell sequencing of human hippocampal tissues from PTSD patients reveals immune dysregulation underpinned by aberrant glial activation and defective neuron–glia crosstalk, identifying disrupted glial–neuronal communication as a core pathogenic driver of PTSD [27]. Our study revealed that diosmin modulates astrocyte phenotypic transition in PTSD models, switching astrocytes from neurotoxic to neuroprotective states. Diosmin suppresses the expression of pro-inflammatory IL-1β and TNF-α and increases levels of anti-inflammatory IL-4 and IL-10, which is consistent with its protective activity against astrocyte injury. Prior investigations in PTSD models have demonstrated that ginsenoside Rg1 and electroacupuncture exert neuroprotective functions by attenuating astrocyte activation and neuroinflammation [28,29].

The molecular underpinning of diosmin’s action appears to involve the suppression of the STAT1-CXCL9 signaling axis. STAT1 is a key transcription factor activated by pro-inflammatory cytokines such as interferon-gamma, and its phosphorylation drives the expression of numerous inflammatory genes, including the chemokine CXCL9 [30]. Our finding that diosmin downregulates both STAT1 and CXCL9 in the hippocampus provides a plausible mechanistic link between its anti-inflammatory effect and the observed astrocytic phenotypic shift. Specifically, inhibiting STAT1 signaling would logically attenuate the transcriptional program promoting the neurotoxic astrocyte phenotype. Furthermore, CXCL9 is not merely a marker but an active player in neuroinflammation; it can recruit cytotoxic T lymphocytes into the brain parenchyma, potentially amplifying neuronal damage [31,32]. By dampening this axis, diosmin may disrupt a feed-forward loop of immune cell recruitment and glial activation. Astrocyte-selective manipulation in vivo would be needed to relate this candidate mechanism to behavior. However, *Stat1* knockdown and diosmin produced overlapping changes in cultured cells, but a nonsignificant combined-treatment comparison does not prove shared-target engagement or that *Stat1* is essential. In addition, the weaker cytokine reductions in the knockdown-containing groups than with diosmin alone warrant consideration of treatment sequence, cell viability, transfection effects, and endpoint-specific regulation.

In our study, the regimen (20 mg/kg, i.p. for 14 days) effectively reversed established PTSD-like behavior. Nevertheless, this study has three notable limitations. First, only a single fixed dose was tested, precluding full characterization of its pharmacological efficacy and therapeutic window. Second, while diosmin-driven amelioration of PTSD-like behaviors is tightly linked to modulation of toxic astrocytic phenotypes and the STAT1-CXCL9 axis, the broader STAT1-governed regulatory network remains incompletely defined. Third, we did not include an SPS&S group receiving 1% DMSO vehicle. Although the existing literature suggests minimal anti-inflammatory activity for 1% DMSO, we cannot fully exclude subtle biological effects of DMSO on glial activation in this PTSD-like mouse model.

## 3. Materials and Methods

### 3.1. Animals and Grouping

Eight-week-old male C57BL/6J mice were purchased from the Laboratory Animal Center of the Air Force Medical University, China (License No. SYXK (Shaan) 2024-003). A total of 24 healthy male mice were randomly assigned to three groups (*n* = 8 per group): Control group, SPS&S group and SPS&S + diosmin group. Prior to the experimental procedures, all mice were acclimatized for one week under standard housing conditions: 12-h light/dark cycle, ambient temperature of 25 ± 1 °C, relative humidity of 50 ± 5%, with free access to standard chow and sterile water. All animal experimental procedures were reviewed and approved by the Laboratory Animal Ethics Committee of the Air Force Medical University (Approval No. 20260001; Dated 26 January 2026).

### 3.2. Establishment of the PTSD Mouse Model

The PTSD model was established using SPS&S protocol, as previously described with minor modifications [33,34]. Briefly, the SPS&S paradigm consisted of four sequential stressors (restraint stress, forced swimming, deep anesthesia, and unconditioned foot shock) administered in the following order: Mice were individually restrained in 50 mL centrifuge tubes (with ventilation holes) for 4 consecutive hours, and immediately after restraint, they were subjected to forced swimming in transparent acrylic buckets (diameter 20 cm, height 30 cm) filled with 25 ± 1 °C water (depth 20 cm) for 20 min. Following a 10-min recovery period, mice were placed in a sealed anesthesia chamber and exposed to 5% isoflurane (gas flow rate: 0.2 L/min) for 10 s, with this anesthesia cycle repeated three times at 5-min intervals; mice were then returned to their home cages for natural recovery (30 min). Subsequently, mice were transferred to a shock chamber (200 × 200 × 250 mm) for a 60-s adaptation period, followed by a single unconditioned foot shock (1 mA, 5 s duration), and post-stimulation, they were allowed to remain in the shock chamber for an additional 30 s before being returned to their home cages with free access to standard chow and sterile water. Mice in the control group were subjected to identical handling procedures (placement in the shock chamber for 60 s) but did not receive foot shock.

### 3.3. Drug Administration

Diosmin powder (Sigma-Aldrich, St. Louis, MO, USA) was first dissolved in dimethyl sulfoxide (DMSO) to prepare a 2 mg/mL stock solution with gentle sonication for complete solubilization. This stock solution was further diluted with sterile normal saline to yield an injection working solution containing a final DMSO concentration of 5% (*v*/*v*). Freshly prepared working solution of diosmin was administered intraperitoneally to mice at a dose of 10 µL/g body weight (20 mg/kg) once daily for 14 consecutive days (Figure 1A). Control mice received an equal volume of sterile normal saline via the same route and schedule.

### 3.4. Behavioral Assessments

#### 3.4.1. Open Field Test (OFT)

The experimental mice were placed in the testing room for 2 h, during which they were allowed to habituate for approximately 3 to 5 min before the commencement of the experiment. Light was dimly illuminated and suitable for mice’s natural exploratory behavior. Mice were placed at the center of a cubic chamber (500 mm × 500 mm × 500 mm) and allowed to travel freely for 15 min. The distance used as a parameter for the locomotion performance was measured with an automated analyzing system (Intelligent Recognition & Communications Biotech Co., Ltd., Suzhou, China).

#### 3.4.2. Elevated Plus Maze (EPM)

The Plexiglas apparatus (Intelligent Recognition & Communications Biotech Co., Ltd., Suzhou, China) consisted of two opposite-facing open arms (300 mm × 50 mm × 50 mm), two opposite-facing closed arms (300 mm × 50 mm × 50 mm), and a central area (50 mm × 50 mm), which was elevated 500 mm above the floor. Each mouse was placed in the central area facing a closed arm at the beginning of the test, and allowed for 5 min of free exploration on the apparatus. The motion trails were recorded with a video camera.

#### 3.4.3. Fear Conditioning and Fear Recall Test

We performed a contextual and cued fear test as described in previous reports [33,34]. The animals were habituated in the experimental room for at least 2 h with the light on before the tests. Before and after each trial, all devices were cleaned with 75% alcohol and paper towels and air-dried. All animals were returned to their home cage after each trial.

For the contextual fear test, we explored the time courses of fear behaviors; after SPS&S 1, 7 and 14 days, the mice were placed in a shock chamber and allowed to freely explore for 300 s and their freezing time was recorded. Subsequently, they were returned to their home cage.

The conditioning and cued fear test protocols were performed. Briefly, on day 17 after SPS&S, the animals were placed in a shock chamber, and the fear conditioning training was performed as previously reported [33,34]. Each animal was allowed to explore the chamber freely for 180 s; then, an auditory cue, comprising a tone of 75 dB, 4500 Hz, was presented for 20 s as a conditioned stimulation (CS) and a foot shock (0.8 mA) performed in the last 2 s of the tone as an unconditioned stimulation (US). This presentation of CS-US pairing was repeated for 6 rounds with 60 s of inter-trial interval (ITI).

On day 18, the animals were placed in a transparent acrylic chamber and allowed to explore the new environment for 2 min followed by a 20-s tone stimulus (75 dB, 4500 Hz). Each mouse underwent six test trials with a total duration of 8 min, and the freezing time throughout the entire procedure was recorded. On day 19, the fear recall test was performed. The mice were placed into the shock chamber without tone or foot shock stimulation. The freezing time of each mouse was recorded over an 8-min observation period.

### 3.5. Immunofluorescent Staining and Sholl Analysis

On day 19 following SPS&S mouse model induction, mice were euthanized via excessive isoflurane and transcardially perfused with 40 mL of 0.01 M PBS, followed by 4% paraformaldehyde for tissue fixation. For primary cell cultures, cells were fixed in 4% paraformaldehyde for 10 min. Both 30 µm coronal hippocampal brain sections and fixed primary cells were subjected to immunofluorescent staining. Samples were incubated with primary antibodies including anti-GFAP (1:500, Abcam, Cambridge, UK), anti-C3 (1:100, Abcam, Cambridge, UK), anti-S100A10 (1:100, Abcam, Cambridge, UK), anti-STAT1 (1:100, Abcam, Cambridge, UK), and anti-CXCL9 (1:500, Abcam, Cambridge, UK), and subsequently probed with anti-mouse/rabbit Alexa Fluor 594/488 secondary antibodies (1:500, Invitrogen, Carlsbad, CA, USA). Fluorescent images were acquired using an FV3000 confocal microscope (Olympus, Tokyo, Japan). ImageJ software (version 1.6.0) was used for auxiliary image analysis. For cell counting, 3–6 sections per hippocampal region from each animal were systematically quantified using ImageJ.

Sholl analysis was used to quantify the amount and distribution of astrocytic arborization at increasing radial distances through the Sholl analysis plugin ImageJ software. Briefly, for each astrocyte, concentric rings centered on the soma were generated starting at 5 µm from the cell body with a 1 µm step size. The plugin quantified the number of branch intersections with these concentric rings. Raw Sholl data were exported and binned at 1 µm intervals in GraphPad Prism (version 9.0).

### 3.6. Western Blot Analysis

Western blotting was performed according to the standard protocol (Molecular Clone, Edition II). Mice were sacrificed after inducing deep anesthesia, and the whole brains were quickly removed. The bilateral hippocampi were collected and homogenized with a hand-held pestle in sodium dodecyl sulfate (SDS) sample buffer. The samples were heated at 100 °C for 10 min and loaded and electrophoresed on 10% SDS-polyacrylamide gels with standard Laemmli solutions. Proteins were electroblotted onto a polyvinylidene difluoride membrane. Membranes were incubated in a blocking solution for 1 h and then incubated overnight under gentle agitation with anti-GFAP (1:500, Abcam, Cambridge, UK), anti-C3 (1:100, Abcam, Cambridge, UK), anti-S100A (1:100, Abcam, Cambridge, UK), anti-STAT1 (1:100, Abcam, Cambridge, UK), anti-CXCL9 (1:500, Abcam, Cambridge, UK), and anti-β-actin (1:5000, Dingguo, Beijing, China). After primary antibody incubation, the membranes were thoroughly washed with TBST buffer to remove unbound primary antibodies, followed by incubation with corresponding secondary antibodies at room temperature. Finally, specific protein bands were visualized using an enhanced chemiluminescence (ECL) detection system, and the band intensity was quantified for the analysis of target protein expression levels using ImageJ (version 1.6.0).

### 3.7. Hippocampal Transcriptome Sequencing

Bilateral hippocampal tissues from six samples (3 SPS&S, 3 SPS&S + diosmin) were rapidly dissected post-euthanasia, snap-frozen in liquid nitrogen, and stored at −80 °C. Total RNA was extracted using the Trizol kit following the manufacturer’s instructions and digested with RNase-free DNase I to remove genomic DNA contamination. RNA quality and integrity were assessed via 1.0% agarose gel electrophoresis, NanoPhotometer spectrophotometer, Qubit 2.0 fluorometer, and Agilent 2100 Bioanalyzer. Validated high-quality RNA was submitted to Sangon Biotech (Shanghai, China) for stranded library preparation and paired-end sequencing on an Illumina platform. Raw reads were trimmed to remove adapters and low-quality sequences. Clean reads were mapped to the mouse reference genome (mm10) using HISAT2; gene expression was quantified with featureCounts, and DESeq2 was applied for differential expression analysis. DEGs were defined as genes with adjusted *p* < 0.05 and |log_2_(fold change)| > 1.

### 3.8. Quantitative Reverse Transcription Polymerase Chain Reaction (qRT-PCR) Analysis

Total RNAs were extracted from the hippocampi of mice using the TRIzol reagent according to the guidelines provided by the manufacturer (Invitrogen). For qRT-PCR, RNA was converted into complementary DNA (cDNA) using the PrimeScript™ RT Reagent Kit (Perfect Real Time, Takara Bio Inc., Kusatsu, Japan), and the reactions were conducted with SYBR^®^ Premix Ex Taq™ (Takara Bio Inc., Kusatsu, Japan) in the CFX96 Touch Real-Time PCR Detection System (Bio-Rad, Hercules, CA, USA). The relative expression levels of genes were assessed using Bio-Rad CFX Manager software, with normalization against the internal control β-actin. The sequences of primers for SybrGreen probes corresponding to the target genes can be found in Supplementary Table S1.

### 3.9. Enzyme-Linked Immunosorbent Assay (ELISA)

The levels of tumor necrosis factor-α (TNF-α), interleukin-1β (IL-1β), interleukin-6 (IL-6), interleukin-4 (IL-4), and interleukin-10 (IL-10) cytokines in mouse serum were quantified using commercial precoated double-antibody sandwich ELISA kits (Beyotime Biotechnology, Shanghai, China), in accordance with the manufacturer’s standard protocols. Briefly, serially diluted cytokine standards and serum samples (100 µL per well) were loaded into pre-coated 96-well plates and incubated at 37 °C for 90 min. After discarding the residual liquid, biotin-conjugated detection antibody working solution was added to each well, followed by a 60 min incubation at 37 °C. The plates were thoroughly washed 3–5 times with washing buffer, and HRP-streptavidin working solution was subsequently applied for a 30 min light-protected incubation at 37 °C. After another round of thorough washing, 3,3′,5,5′-tetramethylbenzidine (TMB) substrate solution was added to initiate chromogenic reaction. The enzymatic reaction was terminated by the addition of stop solution (50 µL per well). The absorbance at 450 nm was detected within 5 min using a microplate reader, and the concentrations of target cytokines in samples were calculated based on the fitted standard curve. All serum samples were assayed in triplicate.

### 3.10. Primary Astrocyte Cultures and siRNA Transfection

Primary astrocyte-enriched cultures were prepared from whole brains of postnatal day 3 mouse pups as previously described [14]. Astrocytes were isolated by orbital shaking, harvested and seeded. After reaching a confluent glial monolayer (12–14 days post-seeding), A1-like astrocytes were induced by 24 h treatment with IL-1β (3 ng/mL), TNF-α (30 ng/mL), and C1q (400 ng/mL), whereas A2-like astrocytes were induced by 24 h exposure to IL-10 (20 ng/mL) and IL-4 (10 ng/mL). Diosmin (20 µg/mL) was then applied to the astrocytes for 6 h. All cellular experiments were performed with at least three independent biological replicates (*n* = 3), each containing three technical replicates.

Chemically synthesized siRNAs were used to knock down mouse *Stat1*. *siStat1*-*1:* sense 5′-CCGAAGAAGUUCACUCUUATT-3′, antisense 5′-UAAGAGUGAACUUCUUCGGTT-3′. *siStat1*-*2:* sense 5′-GCCGAGAACAUACCAGAGAAUTT-3′, antisense 5′-AUUCUCUGGUAUGUUCUCGGTT-3′. *siStat1-3:* sense 5′-GCUGCCUAUGAUGUCUCGUUUTT-3′, antisense 5′-AAACGAGACAUCAUAGGCAGCTT-3′. A scrambled, non-targeting siRNA was included as a negative control (NC). Cells were transfected with 50 nM siRNA or NC siRNA for 24 h using a Transfection Kit (Sangon Biotech, Shanghai, China).

### 3.11. Statistical Analysis

Statistical analyses were conducted in GraphPad Prism. One-way ANOVA with Tukey’s post hoc test was used for multiple-group comparisons. Sholl analysis employed two-way ANOVA followed by Bonferroni-corrected pairwise group comparisons. Two-tailed unpaired or paired Student’s *t*-tests were applied for two-group comparisons. Statistical significance was defined as *p* < 0.05. Differentially expressed genes (DEGs) were screened with thresholds of |log_2_FC| ≥ 1 and adjusted *p* < 0.05.

## 4. Conclusions

In male SPS&S-exposed mice, the diosmin-containing treatment regimen was associated with greater exploration, reduced conditioned freezing, partial recovery of GFAP-labeled hippocampal process measures, and changes in inflammatory markers and STAT1/CXCL9 abundance. Cultured-cell findings nominate STAT1-associated regulation for further investigation but do not establish its necessity for diosmin action.

## Data Availability

The data that support the findings of this study are available from the corresponding author upon reasonable request.

## Supplementary Materials

The following supporting information can be downloaded at: https://www.mdpi.com/article/10.3390/ijms27209342/s1.
