# Supplementary material for: Diosmin Attenuates PTSD-like Behaviors and Hippocampal Astrocyte Alterations with Reduced STAT1 and CXCL9 Expression in an SPS&S Mouse Model
Source: Int J Mol Sci. 2026 Oct 21;27(20):9342. doi: 10.3390/ijms27209342 (PMC13705356; doi:10.3390/ijms27209342)
Supplement: Supplementary file 1 [file ijms-27-09342-s001.zip › ijms-4596621-supplementary.pdf]

Supplementary Table S1

Gene symbol	Forward primer	Reverse primer
stat1	TGCTGTGCCTCTGGAATGATGG	CCTGGCTGCTGGTCCTTGAG
cxcl9	TTGGGCATCATCTTCCTGG	TTGAGGTCTTTGAGGGATTTGTA
Fcgr1	TGAGGTAGCCACGGAGGACAG	ACAGGATGTGAAACCAGACAGGAG
fkbp5	GTGTTGGCAGTCAATCCTCAGAAC	TCGTTGTGCTCCTTCGCCTTC
Fos	TGACTGGAGGTCTGCCTGAGG	CACGTTGCTGATGCTCTTGACTG
Map3k8	AAGTGAAGAGCCAGGAGTGTATGAG	GACAGATGACAGCCAGGGAACC
H2-Q4	ATCCTGACCTGGCAGTTGAATGG	CCAAGAGGCACCACCACAGATG
sphk1	CTGCGGCTCTATTCTGTGCT	GGTGCCCACTGTGAAACG
β-actin	GTGCTATGTTGCTCTAGACTTCG	ATGCCACAGGATTCCATACC
